# Evaluation of Substance P concentrations in the blood plasma of jugular and tail vein of healthy German Simmental cows

**DOI:** 10.1186/s12917-023-03768-0

**Published:** 2023-10-20

**Authors:** Anna Landinger, Yury Zablotski, Gabriela Knubben-Schweizer, Theresa Tschoner

**Affiliations:** Clinic for Ruminants With Ambulatory and Herd Health Services at the Centre for Clinical Veterinary Medicine, LMU Munich, Sonnenstrasse 16, 85764 Oberschleissheim, Germany

**Keywords:** Dairy cattle, Fleckvieh, Pain, Pain assessment, Reference range

## Abstract

**Background:**

Cattle strongly mask their pain, making the recognition and assessment of pain difficult. Different subjective and objective parameters to assess pain have been described. Substance P (SP), which is a neurotransmitter, is used to objectively evaluate nociception in cattle. However, SP concentrations have mainly been described in diseased animals, or animals subjected to painful procedures. To this day, no evaluation of SP in healthy adult cattle has been published.

The objectives of this pilot study were to 1) assess the SP concentrations in healthy adult German Simmental cattle in the blood plasma, 2) compare the concentrations between the blood of the jugular and the tail vein, and 3) assess the concentrations in the blood of the tail vein every 6 h over a period of 24 h.

A total of 52 healthy cattle of the German Simmental breed were included in this study. Animals were 5.0 ± 1.3 (mean ± SD) years old and between 117 and 239 (175.0 ± 34.1) days in milk. Blood samples were taken from the jugular vein (BJV, 07:45 a.m.) and the tail vein (TV1, 08:00 a.m.) each. Additional blood samples were taken every 6 h over the course of 24 h from the tail vein (TV2 – TV5). SP concentrations were analyzed using a commercial ELISA kit.

**Results:**

Mean (± SD) and median SP concentrations were 1.087 ± 436 pg/ml and 984 pg/ml for BJV (range 502 – 2,337 pg/ml), and 920 ± 402 pg/ml and 818 pg/ml for TV1 (range 192 – 2,531 pg/ml), respectively. There was a significantly positive correlation between SP concentrations of BJV and TV1. SP concentrations between BJV and TV1 were significantly different, as were SP concentrations in the tail vein between sampling times over the course of 24 h.

**Conclusions:**

The results of this study show that blood samples to assess SP concentrations in cattle can be taken from the jugular as well as from the tail vein. There are high variations in concentrations between animals, and it is hard to define reference ranges for SP in healthy animals. Repeated blood samples should not be taken by repeated punctation of a vein but by use of a jugular vein catheter, which is a major limitation of the present study.

**Supplementary Information:**

The online version contains supplementary material available at 10.1186/s12917-023-03768-0.

## Background

According to Molony and Kent (1997), pain is “an aversive sensory and emotional experience representing an awareness by the animal of damage or threat to the integrity of its tissues. It changes the animal’s physiology and behavior to reduce or avoid the damage, to reduce the likelihood of recurrence and to promote recovery” [1].

The assessment of pain can be a challenge in cattle, as these animals strongly mask their pain and hide obvious pain and discomfort; masking pain is a defense mechanism to not be perceived as weak by a predator [2]. Assessment of painful states in cattle can be approached by subjective or objective methods [2, 3].

For years, cortisol has been used as an objective biomarker for the assessment of pain in cattle. However, the use of cortisol has its limitations; not only acute pain-related stress, but also human presence, handling, and restraint [4], as well as individual and anxiety related behavior [5] and different external environments and management techniques [6] result in changes of cortisol concentrations. Also, cortisol concentrations show a circadian rhythm [7], which is another limitation for using cortisol as an indicator for pain.

Substance P (SP) is a sensory neurotransmitter, belonging to the family of tachykinins, and is composed of 11 amino acids [8]. SP is released both from the central and the peripheral nervous system and interacts with neurokinin receptors during painful states [9]. In 2008, Coetzee et al. showed that cortisol concentrations increased both in castrated and sham-castrated calves, whereas SP only increased in the castrated animals, indicating that SP might be a more sensitive and objective indicator for nociception and pain than cortisol [10].

Research about the use of SP as a biomarker for pain in bovine medicine mainly focused on the evaluation of plasma Substance P concentrations (PSPC) in animals which were exposed to a painful as well as stressful event. Changes in concentrations of SP in blood plasma or serum during castration [10], dehorning [11], transportation [12], or umbilical surgery [13] have been described. Additional studies also examined plasma SP concentrations in animals suffering from inflammation or infection which presented as clinical metritis [14] or lameness [15]. Throughout these studies, SP concentrations of cattle show high inter- and intraindividual variances [10, 13]. However, basic research work about SP concentrations in cattle is missing.

Studies investigating SP concentrations in healthy adult cattle, which are neither exposed to a painful nor to a stressful environmental influence, are rare. However, if physiologic or reference ranges of SP concentrations could be established, this could support researchers in objectively differentiating between sound animals and animals in pain.

Therefore, the aims of the present pilot study were to 1) evaluate the PSPC in the blood of healthy cows of the German Simmental breed, 2) compare the concentrations between blood taken either from the jugular or the tail vein, and 3) to evaluate PSPC after multiple puncturing of the tail vein.

## Results

### Animals

Age, number of lactations, days post-partum, stage of pregnancy, and daily milk yield of the study animals are presented in Table 1. Parameters in all cows are given in Appendix 1.

**Table 1:** General findings, findings of clinical examination according to Dirksen et al. (1979), and laboratory findings of selected blood parameters in 52 adult cows sampled for the evaluation of substance P concentrations in healthy German Simmental cattle. Parameters are presented as mean and standard deviation (SD). Ranges are given in brackets

Parameter	Mean	SD
**General Findings**		
Age in years	5.0 (3.4 – 9.1)	1.3
Number of lactations	3.2 (2.0 – 7.0)	1.3
Days post-partum	175.0 (117 – 239)	34.1
Days pregnant	65.7 (39.0 – 166.0)	29.6
Milk yield (kg/day)	37.5 (29.3 – 51.6)	4.9
**Findings of Clinical Examination**		
Temperature (°C)	38.4 (38.0 – 39.1)	0.3
Heart Rate (beats/minute)	82.1 (68 – 100)	6.0
Respiratory Rate (breaths/minute)	34.1 (20 – 48)	7.2
Rumen Cycles (in 2 min)	2.3 (1 – 3)	0.7
**Findings of Laboratory Analysis**		
Leycocyte Count (4 – 10 × 10^3^/µl)	7.0 (4.0 – 12.7)	1.7
Packed Cell Volume (30 – 36%)	32.4 (26.5 – 41.6)	3.1
Hemoglobin (10 – 13 g/dl)	10.3 (8.3 – 13.6)	1.1
Total Protein (40 – 80 g/l)	74.8 (67.0 – 83.6)	4.0
Glutaraldehyde Test^1^ (> 15 min)	15.9 (14 – 16)	0.4
Beta Hydroxybutyrate (< 1.2 mmol/l)	0.62 (0.34 – 0.98)	0.16
Non-Esterified Fatty Acids (< 0.39 mmol/l)	0.11 (0.03 – 0.30)	0.04
Glutathione Peroxidase (> 250 U/gHb)	610.0 (368 – 828)	108.3
^1^Glutaraldehyde Test is missing in 1 animal		

### Clinical examination

Findings of clinical examinations are presented in Table 1. All animals included in the present study were clinically healthy. Results of rectal temperature, heart and respiratory rate, rumen activity, and examination of the consistency of the feces are given in Appendix 1.

### Laboratory findings

Laboratory findings are presented in Table 1. Laboratory findings were within the reference ranges determined by the Clinic for Ruminants with Ambulatory and Herd Health Services in 40.4% (*n* = 21) of animals. Fecal samples were negative in all animals. Laboratory findings of all 52 animals are given in Appendix 2.

### Plasma substance P concentrations in jugular and tail vein

**Fig. 1:**
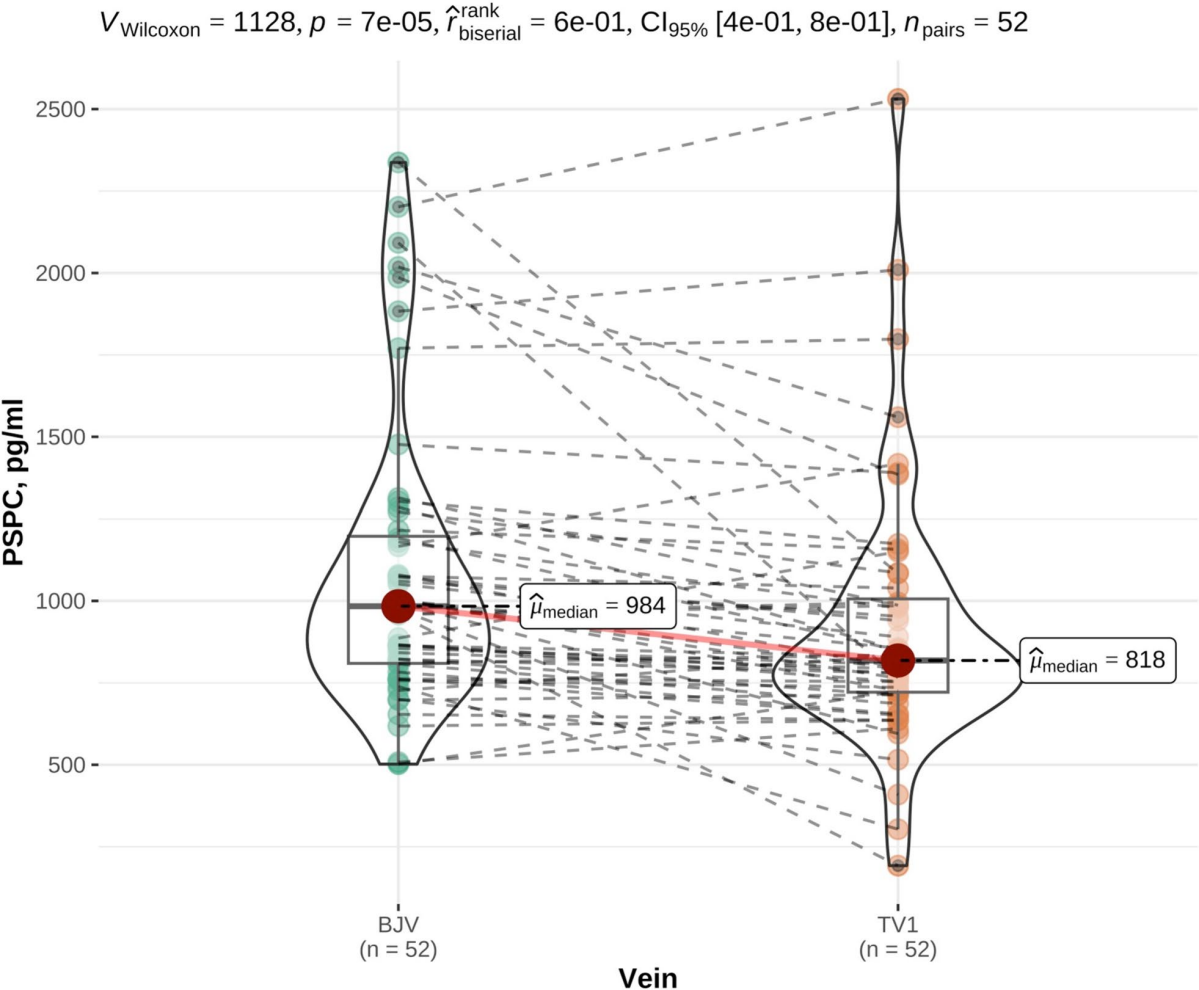
Comparison of plasma substance P concentration (PSPC) in the blood taken from the jugular and tail vein. PSPC were assessed from the Vena jugularis (jugular vein, BVJ) and the Vena caudalis mediana (tail vein, TV1) of 52 clinically healthy cattle of the German Simmental breed. Samples were taken at 7:45 a.m. and 08:00 a.m. (jugular and tail vein, respectively). Concentrations between BJV and TV1 did differ significantly (p < 0.01). Dotted lines link the PSPC in the blood samples taken from the jugular and tail vein of individual animals

PSPC determined from the blood taken from the jugular and tail vein are presented in Table 2. PSPC in all individual animals are given in Appendix 3. PSPC were significantly higher in BJV compared with TV1 (median 984 pg/ml and 818 pg/ml respectively, *p* < 0.01, Fig. 1). There was a significantly positive correlation (*p* < 0.01, Spearman correlation coefficient (rho) = 0.76) between BJV and TV1. PSPC did not differ significantly between feeding groups 1 and 2, neither in BJV (*p* = 0.06), nor in TV1 (*p* = 0.9, Table 3). Also, there was no significant difference in PSPC in clinically healthy animals with physiological (PHYS) compared with non-physiological (MDEV) laboratory findings both in BJV (*p* = 0.8) and TV1 (*p* = 0.8) (Table 3).

**Table 2:** Plasma substance P concentration (PSPC) in the blood from the Vena jugularis (Jugular vein, BVJ) and Vena caudalis mediana (Tail vein, TV1 to TV 5) of 52 adult cows sampled for the evaluation of substance P concentrations in healthy German Simmental cattle. Blood samples were taken at 07:45 a.m. (BJV), 08:00 a.m (TV1), 02:00 p.m. (TV2), 08:00 p.m. (TV3), 02:00 a.m. (TV4), and 08:00 a.m. (TV5). Blood samples taken from the tail vein were missing for *n* = 2 in TV2, *n* = 4 in TV3, and *n* = 5 for TV 4 and TV5, respectively. Parameters are presented as mean with standard deviation (SD), and median. Ranges are given in brackets. PSPC between BJV and TV1 differed significantly (*p* < 0.01)

PSPC (pg/ml)	Mean	SD	Median
**BJV** (502 – 2,337)	1,087	436	984
**TV1** (192 – 2,531)	920	402	818
**TV2** (497 – 2,512)	977	412	842
**TV3** (168 – 2,293)	1,003	425	953
**TV4** (415 – 2,974)	1,135	441	1,024
**TV5** (458 – 3,360)	1,088	498	943

**Table 3:** Plasma substance P concentration (PSPC) in the blood from the Vena jugularis (Jugular vein, BVJ) and Vena caudalis mediana (Tail vein, TV1) of animals according to feeding group and laboratory findings. Animals were kept in two different feeding groups (groups 1 (*n* = 18) and 2 (*n* = 24)) due to research purposes. PSPC between feeding groups did not differ significantly. According to laboratory findings, animals were either grouped as PHYS (21 clinically healthy cows with laboratory findings within the reference ranges) and MDEV (31 clinically healthy cows with mild deviations from the reference ranges). There were no significant differences in PSPC between PHYS and MDEV. Parameters are presented as mean with standard deviation (SD), and median. Ranges are given in brackets

	**PSPC (pg/ml)**		
	**Mean**	**SD**	**Median**
**Feeding Groups**			
**BJV**			
**Group 1** (502 – 1,303)	90	210	875
**Group 2** (508 – 2,337)	1,185	493	1,026
**TV1**			
**Group 1** (636 – 1,174)	868	174	832
**Group 2** (192 – 2,531)	948	482	802
**Groups According to Laboratory Findings**			
** BJV**			
**PHYS** (508 – 2,019)	1,041	387	1,000
**MDEV** (502 – 2,337)	1,119	471	961
**TV1**			
**PHYS** (303 – 1,560)	886	333	806
**MDEV** (192 – 2,531)	944	446	833

### Plasma substance P concentration in the tail vein over the period of 24 h

**Fig. 2:**
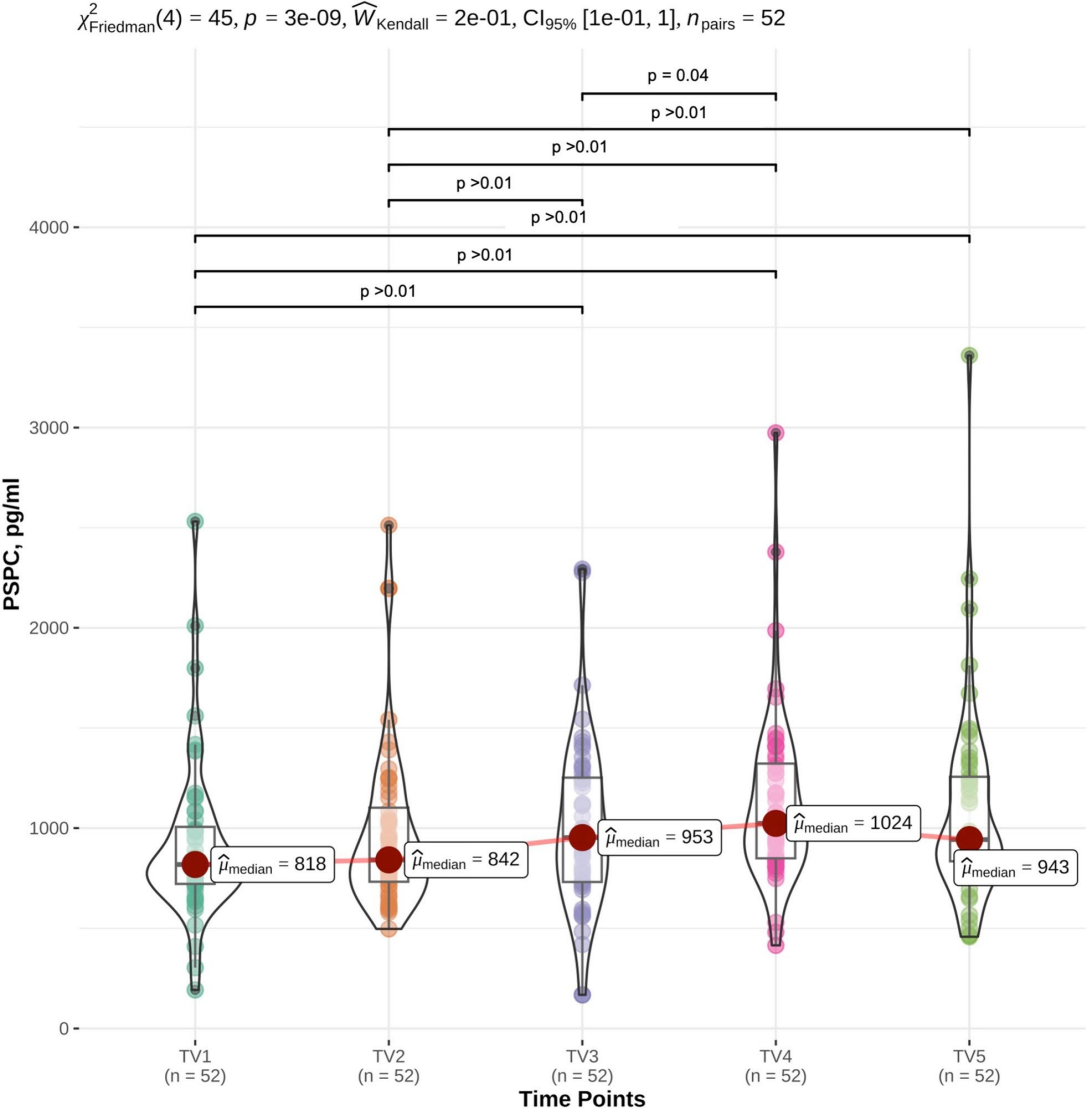
Course of plasma substance P concentration (PSPC) in the blood of the tail vein over the course of 24 h. Blood for the assessment of PSPC was taken from the Vena caudalis mediana (tail vein) of 52 adult cows at the interval of 6 h over the course of 24 h (08:00 a.m. (TV1), 02:00 p.m. (TV2), 08:00 p.m. (TV3), 02:00 a.m. (TV4) and 08:00 a.m. (TV5). Blood samples were missing for *n* = 2 in TV2, n = 4 in TV3, and *n* = 5 for TV 4 and TV5, respectively. The missing values were imputed via chained random forest approach. Significant differences in PSPC between blood samples are given as *p* < 0.05 and *p* < 0.01

Mean, SD, and median of PSPC in the tail vein over a period of 24 h are given in Table 2. Differences in concentration are presented in Fig. 2. There were significant differences between TV1 and TV3 (*p* < 0.01), TV1 and TV4 (*p* < 0.01), TV1 and TV5 (*p* < 0.01), TV2 and TV3 (*p* < 0.01), TV2 and TV4 (*p* < 0.01), TV2 and TV5 (*p* < 0.01), and TV 3 and TV4 (p = 0.04).

### Plasma substance P concentration in correlation to different parameters

There was no correlation between PSPC and THI, neither for BJV, nor for TV1 (*p* = 0.49, rho = 0.10 and *p* = 0.39, rho = 0.12, respectively). There was no correlation between PSPC and age of animals, neither for BJV, nor for TV1 (*p* = 0.75, rho = -0–05 and *p* = 0.88, rho = -0.02, respectively). There was no correlation between PSPC and lactation number of animals, neither for BJV, nor for TV1 (*p* = 0.62, rho = 0.07 and *p* = 0.82, rho = -0.03, respectively). There was no correlation between PSPC and days in milk of animals, neither for BJV, nor for TV1 (*p* = 0.51, rho = 0.09 and *p* = 0.63, rho = 0.07, respectively). There was no correlation between PSPC and heart rate of animals, neither for BJV, nor for TV1 (*p* = 0.27, rho = 0.16 and *p* = 0.82, rho = 0.03, respectively).

## Discussion

The present study was conducted to evaluate the PSPC in the jugular and tail vein of healthy, adult German Simmental cows. The study population is representative of cows in Bavaria, Germany.

### Clinical examination, and laboratory findings

Changes to PSPC are not only dependent on the experience of pain [10], but also on inflammatory status [16, 17], and stress [18–20]. To include more animals into the statistical model, clinically healthy animals with only minor deviations from laboratory findings were not excluded from the present data set. This could be seen as a confounder; however, reference ranges always include a 5% false-positive rate [21], and minor differences from reference ranges are less likely to indicate disease. Differentiating between healthy and diseased animals based on laboratory findings can be difficult, with deviations from defined reference ranges occurring in 2 to 24% of clinically healthy cattle, depending on the variable [22]. To exclude the influence of pain on the present study population, only animals which were clinically healthy were included in this study. To exclude subjectivity in the clinical examination as best as possible, the examination was done as described by [23], and by two of the authors (AL was trained by TT), so that inter-observer-bias is assumed to be minimal. Blood samples were taken during all seasons, which might have influenced physiological parameters [24, 25], but as no animal was excluded due to increased heart rate, respiratory rate, or increased rectal temperature, seasonal influence on the selection of animals can most likely be excluded.

### Plasma substance P concentrations in jugular and tail vein

Overall, PSPC in the present study are distinctively higher than concentrations published in other studies, e.g. 260 to 420 pg/ml in healthy heifers [26], 250 ± 90 pg/ml in non-lame cows [15], 37.73 ± 5.41 pg/ml in cows following parturition [14], and 93.4 ± 17.2 pg/ml in bulls [27]. It is difficult to compare the present data with other publications, as animals in other studies were either diseased [14], diseased and treated with analgesics [28, 29], or submitted to painful [30, 31], or stressful [12, 27] procedures. An overview of PSPC in adult cattle in the existing literature can be found elsewhere [32]. In the present study, PSPC in blood taken from the jugular vein was significantly higher compared with PSPC in the blood taken from the tail vein. As the blood sample from the tail vein was taken after the blood sample from the jugular vein, the authors would have expected the PSPC in the blood from the tail vein to be increased due to the pain caused by punctation. Studies showed that SP results in a slow response, with a delayed onset of excitation of the dorsal horn of 20 to 40 s, and a slow response which lasts 30 to 90 s [33–35]. The time difference between sampling in our study protocol was 15 min; therefore, increase of PSPC in the blood from the tail vein due to pain should have been obvious. Due to the anatomical structures and the small lumen of the Vena caudalis mediana, it is possible that blood samples collected at the tail vein included venous, as well as arterial blood. There are no studies describing possible differences of PSPC in venous compared with arterial blood. In dairy cattle, differences in concentrations of variables (concentrations of insulin, glucose, NEFAs, BHB, and urea) measured in blood taken from either the jugular or the mammary vein were reported [36]. Significant differences in mean values of pCO2, pO2, HCO3-, and BE were found between arterial blood samples taken from the Arteria axillaris and the Arteria auricularis caudalis [37]. Therefore, differences in PSPC between BJV and TV1 could have been caused by the location of the puncture site.

Another possible explanation for the difference in PSPC between blood samples could be different factors associated with sample handling [38]. SP concentrations analyzed from samples which were kept in an ice bath were significantly (*p* < 0.005) higher compared with SP concentrations from samples kept at ambient temperatures [38]. For the present study, all samples were put in an ice bath right after sampling and centrifuged within two hours. The only handling difference BJV and TV was the timing of adding the protease inhibitor. For BJV, aprotonin was added to the EDTA tubes before sampling, whereas for TV1 to TV5, aprotonin was added right after blood sampling with vacutainer systems, within a time span of 1 to 2 min. We cannot exclude that this short time lag caused some small differences in SP concentrations. However, as [38] found that there were no significant differences in total immunoreactivity of SP among samples treated different enzyme inhibitors (including EDTA) if processed immediately, it is therefore highly unlikely that the respective time lag in adding aprotonin influenced the findings of the present study.

### Plasma substance P concentrations over the period of 24 h

For the present study, PSPC was assessed every 6 h over a time span of 24 h to investigate the effect of multiple puncturing of a vein on PSPC, and to assess if PSPC could be subject to a circadian rhythm, as described for cortisol [7]. Our data show that PSPC increased within the 24-h sample period; PSPC differed significantly even between samples taken at the same time 24 h apart (TV1 and TV5). These results indicate that PSPC increases if samples are taken by repeated punctation of the same vein, possibly due to pain. In human medicine, SP is described to be an inflammatory marker [39]. In the course of an inflammation, SP concentrations in the sensitive neurons and in the central nervous system increase. The chemotaxis of neutrophil and eosinophil granulocytes, as well as the migration of cells to the site of inflammation, is controlled by SP [16, 17, 40, 41]. Therefore, PSPC could also have increased due to a local inflammation of the tail vein, caused by the repeated insult to the tissue. The tail vein was only assessed visually for signs of inflammation, and we did not repeatedly assess any other inflammatory markers. Therefore, an evaluation of a possible state of inflammation cannot be made. Additionally, PSPC are subject to stress. According to studies done in laboratory animals, SP was released into the uterine tissue of pregnant mice following the exposition to a stressor [18, 19, 42]. As blood samples were taken repeatedly every 6 h and cows were either caught in a feeding fence or in their cubicles by two people, it is possible that this handling resulted in an increased stress level and therefore, increased PSPC. To exclude the influence of pain, inflammation, and stress on the PSPC, repeated blood samples should have been taken using a jugular vein catheter. Due to the study design (keeping the animals in their housing, feeding, and management system) this was not possible, which is a major limitation of the present study. Also, the study design of the present study was not suitable to investigate a circadian rhythm of PSPC. To assess changes of PSPC during shorter timespans over the course of 24 h, a higher number blood samples should have been taken in shorter intervals of time (e.g. 15 min) over a longer time span and at weekly intervals, as described by [7] for cortisol concentration.

### Plasma substance P concentrations in correlation to different variables

Heat stress was a factor which could have influenced the present results, as heat stress weakens SP-immunoreactivity in chromaffin cells of the adrenal medulla of rats [43]. As there was no significant correlation between THI and PSPC, neither for BJV nor for TV1, influence of heat stress on our results can be neglected for the present data set. Increased heart rates in some animals indicated that animals were stressed due to the study design. External as well as internal stress results in significantly increased heart rates in cattle [44]. However, there was no significant correlation between heart rate and PSPC. To limit the influence of stress on PSPC, animals were not taken from their familiar surroundings for the duration of the study. Regrouping results in a significant increase of saliva cortisol levels in calves [45]. Studies on the influence of regrouping stress on SP are missing. However, previous research showed that PSPC did not increase in animals submitted to a laparoscopic abomasopexy, in contrast to cortisol concentrations [31]. Therefore, and as animals were only regrouped into smaller groups within their group and in their barn, the stress of regrouping is considered to have no major influence on the results of the present study. Age of an animal also seems to have an influence on PSPC. Dockweiler et al. [46] found that PSPC were significantly higher in 6-months old compared with 8-week-old calves following castration. As we did not find any correlation between PSPC and age of the cattle in our study population, it is possible that the age difference in PSPC can be neglected in older cattle. However, parity seems to have an influence on PSPC. Barragan et al. [47] stated that circulating SP concentrations were significantly (*p* = 0.04) higher in primiparous, compared with multiparous cows. As only multiparous cows (two or more calvings) were included into the present study, the authors cannot assess the influence of parity on PSPC. However, there was no correlation between number of lactation and PSPC in our data set. To exclude any possible influence of a pregnancy on PSPC, only pregnant cattle were sampled for the present study. Another factor which should be considered is the temperament of the animals. Kasimanickam et al. [48] found that prior to weaning and breeding, excitable cattle showed significantly higher SP concentration compared with calm cattle. Therefore, temperament could explain the high variability in PSPC between individual animals. Animals in the present study were grouped according to feeding rations. Cows in feeding group 1 were fed concentrates according to their milk yield, whereas cows of feeding group 2 were part of another research project and were fed with a total mixed ration [49–52]. It is possible that the different amounts of concentrates fed to the animals could have influenced PSPC. Subacute ruminal acidosis, which can be caused by feeding excessive amounts of concentrates, results in an inflammation with an increase in acute phase proteins [53]. SP concentrations increase during an inflammation [16, 17, 40, 41]. As PSPC did not differ significantly between feeding groups, the influence of feeding rations was neglected for the present study.

### Method of sampling and analysis

One major limitation of the present study is that blood samples for assessment of PSPC were taken using punctation of a vein, which is a painful procedure. Using saliva samples, a non-invasive procedure, to assess PSPC, has been described by Mayer (2019) [54]. However, Jasim et al. (2018) found significant variations between plasma and salivary SP concentrations in humans [55], and Mayer (2019) found no correlation between plasma and salivary SP concentrations [54]. Also, aprotonin, which is an enzyme inhibitor and keeps SP from degrading [38], can only be added after collection and processing of the saliva samples in the laboratory [54], which was not feasible for the present study due to the study design. Therefore, this sampling method was rejected for the present study. In a review about SP, Snijdelaar et al. (2000) state that SP is involved in slow pain transmission, and that other neurotransmitters are responsible for the fast transmission of pain [9]. Therefore, blood sampling by punctation of the jugular vein was preferred over the use of saliva samples. Different methods of sample handling can influence SP concentration [38]. For the present study, a multispecies ELISA kit was used. The intra- and interassay coefficient of variation was high. However, this ELISA kit has been used in previous studies with good results [13, 31, 56]. Other authors described analysis of PSPC in adult cattle using competitive radioimmunoassay techniques [14, 47] or competitive immunoassays [30]. A high between- and within-animal variation in SP concentration, which was found in the present study, has already been described in adult cattle [31], as well as in calves [10]. As described for cortisol [6], management techniques, as well as external environment, could have an influence on PSPC. As all animals of the present study population were subject to the same handling, management, and environment, these factors can be excluded.

### Establishment of benchmarks of orientation for plasma substance P concentrations

Reference ranges normally include 95% of the concentration/values from normal animals [21]. A major limitation of the present study is that, even though we assessed PSPC in clinically healthy animals, we are not able to provide proper benchmarks of orientation for PSPC. PSPC had a high within- and between animal variability, which is in accordance with previous research work [10, 31]. Also, even though we tried to include only clinically healthy animals, cows showing minor deviations from reference ranges in laboratory findings might have influenced our results, even if there was no significant difference between groups PHYS and MDEV. For the present study, we only assessed PSPC with one assay kit, which limits the use of our data.

## Conclusions

SP is described to be a biomarker for pain in cattle, but basic research work is missing. Our results show that PSPC concentrations taken from the jugular and the tail vein within a time difference of 15 min differ significantly. If single blood samples for the assessment and discussion of PSPC are taken, the investigating person should be aware that PSPC are probably lower in blood taken from the tail compared with the jugular vein. Also, repeated puncture of the tail vein results in an increase of PSPC, indicating that inflammatory processes might have an influence on PSPC in the blood plasma of cattle. Therefore, blood samples should be taken by means of a jugular vein catheter, if repeated measures are necessary, e.g., for research purposes, to minimize an interference of study results.

### Material and methods

All experimental procedures in the present study were approved by the ethics committee of the government of Upper Bavaria (reference number 55.2–1-54–2532-12–13). All study animals were housed at the Research- and Teaching Center Achselschwang, Germany, which is part of the Bayerische Staatsgüter (state-owned enterprise). The present study was conducted as a pilot study. According to literature, samples sizes for pilot studies are recommended to be between 10 to 12 and 15 [57–59] animals. Sample collection was done from December 2019 to June 2021, to include as many healthy adult cows as possible. In this period of time, all adult cattle eligible for this study were included for data collection.

### Cows, housing, and feeding

A total of 52 multiparous cows of the breed German Simmental (at least ≥ 75%) aged 5.0 ± 1.31 years (3.4 – 9.1 years) were included in this study. Animals were 117 to 239 days in milk (175.0 ± 34.1 days). Number of lactations was 3.2 ± 1.3 (2 – 7 lactations), with an average daily milk yield of 37.5 ± 4.9 kg (29.5 – 51.6 kg).

Cows were kept in loose housing on solid ground with deep bedded cubicles and were milked twice daily. Functional claw trimming was performed three times a year.

Cows were fed in three feeding groups according to their milk yield/performance, resulting in study animals originating from two different feeding groups (groups 1 and 2). Group 1 animals (34.6%, *n* = 18) included high yielding animals with a total mixed ration (including gras and maize silage, hay, and straw) and concentrates on demand via transponder. Animals in group 2 (65.4%, *n* = 34) were included in feeding studies conducted by the Bavarian State Research Center for Agriculture and were fed with a total mixed ration with a concentrate ratio of 38.1% of the dry matter [49–52]. These feeding studies did not have any influence on the present study, as animals were kept in their familiar surroundings for the duration of the collection of samples for this study.

**Fig. 3:**
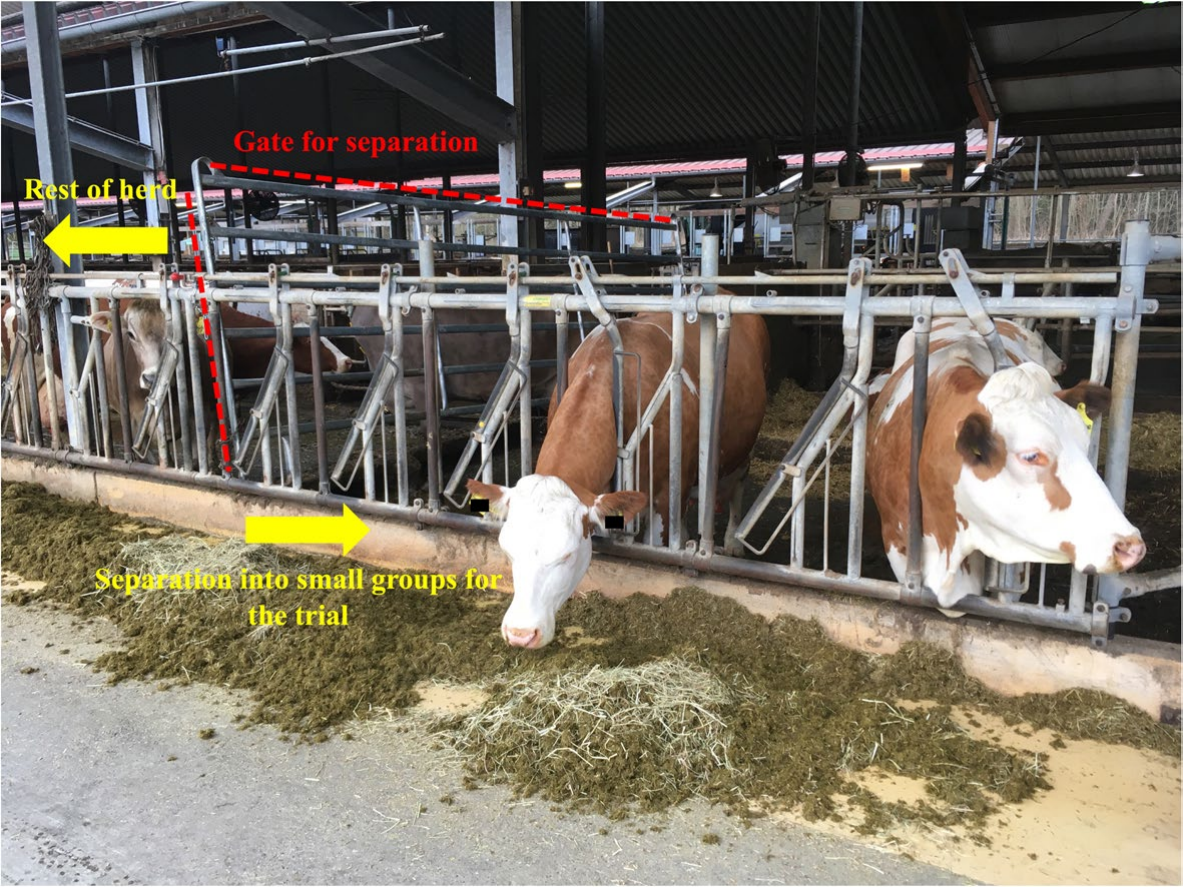
Housing of study animals during the duration of the trial. Animals were housed according to feeding groups. Animals of group 1 (*n* = 18) were separated into a small area of the stable, with visual and tactile contact to the rest of the herd. Animals of group 2 (*n* = 34) remained in the herd for the duration of the study

For the collection of the blood sample from jugular and tail vein at 07:45 a.m. and 08:00 a.m. and the following clinical examination, all cows were caught in a feeding fence for easier management and handling, Afterwards, animals of group 1 were then kept in a fenced off area of the stable (Fig. 3) with visual and tactile contact to the rest of the herd. Animals of group 2 remained in their group for the whole duration of the study.

### Inclusion criteria

At the time of sampling cows had to be at least in their second lactation and pregnant (day 60 to 250 of lactation). Cows had to be clinically healthy, with no treatment with an antibiotic or analgesic drug within the last 14 days before the start of the trial. Clinical examination was done as described by [23] on day 1 (D1) of the trial by one of two authors (AL was trained by TT). Cows were excluded from the study if they were diagnosed with a disease and/or treated as stated above within seven days following the time of blood sampling. To better screen the health status of cows, defined blood parameters (concentrations of leucocytes, hemoglobin, packed cell volume (PCV), selenium, total protein, beta hydroxybutyrate (BHBA), non-esterified fatty acids (NEFAs), and glutaraldehyde test), as well as fecal samples (eggs of *Fasciola hepatica*, *Paramphistomum* spp., gastrointestinal helminths, and larvae of *Dictyocaulus viviparus*) were analyzed, and animals were excluded from the study if these parameters were not within physiological ranges. A total of 77 adult cows were submitted to blood sampling for this study, with only 21 cows showing no deviations from the references ranges for blood parameters used at the Clinic for Ruminants with Ambulatory and Herd Health Services. To be able to include more animals into the statistical model, clinically healthy cattle were included if the laboratory findings were within the following ranges (references ranges as defined by the clinic for ruminants are given in brackets): 3 – 15 × 10^3^/µl (4 – 10 × 10^3^/µl) for leucocyte count, 8 – 15 g/dl (10 – 13 g/dl) for hemoglobin, 26 – 42% (30 – 36%) for PCV, 40 – 85 g/l (40 – 80 g/l for total protein, and > 14 min (> 15 min) for glutaraldehyde test. Animals were divided into either PHYS (no deviations from the reference ranges (40.4%, *n* = 21), or MDEV (clinically healthy with mild deviations from the reference ranges (59.6%, *n* = 31)).

### Collection of blood samples

**Fig. 4:**
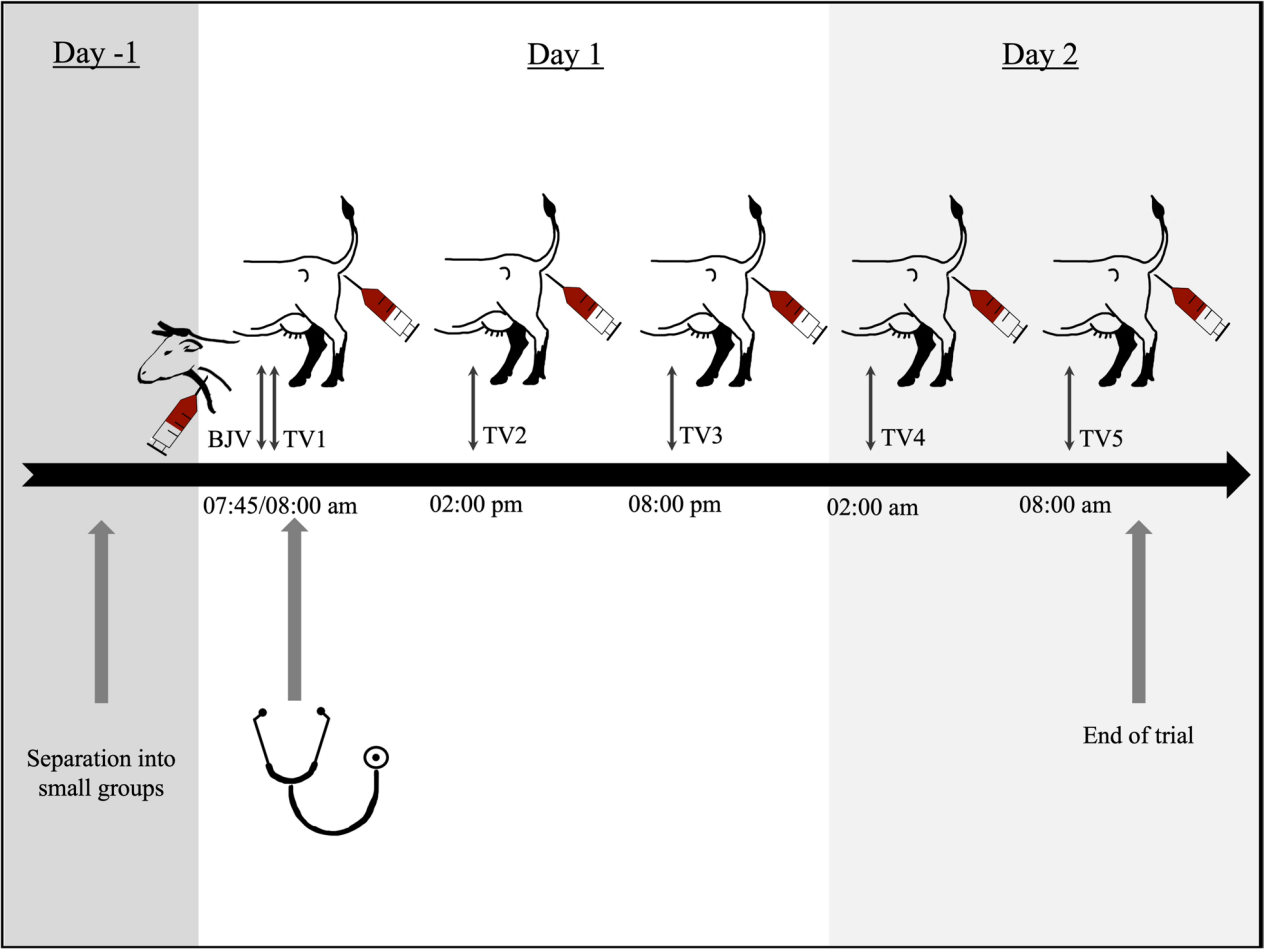
Study protocol for blood sampling in cows for the trial Blood samples were taken over a period of two days. For the basal substance *P* concentration (BJV) sampling time was at 7:45 a.m. of the first study day (D1) from the Vena jugularis. For the determination of a circadian rhythm of substance *P* additional blood samples (TV1—TV5) were taken from the Vena caudalis mediana with intervals of six hours (8:00 a.m., 02:00 p.m., 08:00 p.m. on d1 and 2:00 a.m. and 8:00 a.m. on day 2 (D2) of the study). Clinical examination and fecal sampling were performed after sampling of the BJV and TV1 at 8:15 a.m. on D1

Blood sampling was done over a period of 48 h and samples were taken according to a defined schedule. The study protocol is given in Fig. 4. For blood sampling from the Vena jugularis, cows were caught in a feeding fence and tethered with a halter with the neck bent to one side.

On day 1 of the trial, four blood samples were taken at 7:45 a.m. at the Vena jugularis with a 14G syringe. One sample was collected in a 10 ml EDTA tube containing 45 μl aprotonin per tube for analysis of SP (BJV, basal concentration of SP). The remaining three samples were taken for analysis of concentrations of leucocytes (whole blood), hemoglobin (whole blood), PCV (whole blood), glutathione peroxidase (whole blood), total protein (serum), NEFA (serum), BHBA (serum) and glutaraldehyde test (whole blood).

At 08:00, another blood sample was taken at the Vena caudalis mediana (TV1) with the animals still caught in the feeding fence. Further blood samples to investigate the daily rhythm of substance P in the blood plasma were taken at the Vena caudalis mediana at 02:00 p.m. and 08:00 p.m. on d1, and 2:00 a.m. and 8:00 a.m. at day 2 (D2) of the trial (TV2 to TV5, respectively) with cows either fixated in the feeding fence, or else in their cubicles.

For blood sampling at the Vena caudalis mediana, the bottom of the tail was cleaned and disinfected with alcohol. Blood samples were taken with a 20G syringe and a vacutainer system (4 ml EDTA tubes). After blood was collected, 18 μl aprotonin was added immediately to the EDTA tubes.

During blood sampling, samples for the determination of PSPC were kept on ice at all times, and were centrifuged (4 °C, 1600 × g for 15 min) within two hours after blood collection at the Research- and Teaching Center Achselschwang. Blood plasma for analysis of SP was kept frozen at -20 °C at the Research- and Teaching Center Achselschwang and at the Clinic for Ruminants with Ambulatory and Herd Health Services until the end of the experimental period. Serum samples were centrifuged (4 °C, 1000 × g for 10 min) and further processed at the Research- and Teaching Center Achselschwang. Serum, bloodgas, EDTA, and fecal samples were stored in a refrigerator until the following day, on which these samples were transferred to the Clinic for Ruminants with Ambulatory and Herd Health Services for analysis. Blood samples were analyzed using the Vetscan® HM5 (Abaxis, Union City, USA) for plasma samples, the RAPIDPoint® 450 (Siemens, Munich, Germany) for whole blood samples kept in a bloodgas tube, and the cobas® c 311 Analyzer (Roche, Basel, Switzerland) for serum samples and glutaraledyde test. Faecal samples were evaluated microscopically following sedimentation (fasciolosis, paramphistomosis), flotation (gastro-intestinal helminths), and the Baermann method (dictyocaulosis).

### Temperature humidity index

To investigate the effect of heat stress on PSPC, temperature humidity index (THI) was assessed for each sampling time. Temperature (°C), relative humidity, and THI were recorded every 10 min using a climate sensor (smaXtec animal care, Graz, Austria) which was positioned at the Research- and Teaching Center. Data recorded at 07:40 a.m. (BJV) and 08:00 a.m. (TV1) were included in this study. Due to technical reasons, data for one animal is missing.

### Substance P analysis

SP concentrations in blood plasma were determined using a SP ELISA kit (Enzo Life Sciences GmbH, Lörrach, Germany) as described previously [13, 31]. Optical densities were assayed in duplicate, and means were generated for the calculation of concentrations. Lower and upper limits for the quantification of the SP ELISA kit were 167.78 pg/ml and 1,000 pg/ml. The intra- and interassay coefficient of variation was 20%. According to the manufacturer, the ELISA kit comes with a sensitivity of 5.3 pg/mL.

### Statistical analysis

Data analysis was performed using R 3.6.3 (2020–02-29). Results with a *p*-value < 0.05 were considered statistically significant. One sample each of three animals were excluded from the statistical analysis as PSPC were below detection limit of 167.78 pg/ml. A total of 16 missing values (2 for TV2, 4 for TV3, 5 for TV4 and 5 for TV5) were imputed via chained random forest approach [60]. Normality of data was accessed via Shapiro–Wilk normality test and visually via Quantile–Quantile Plots. Due to a not-normally distributed data a nonparametric Spearman correlation was conducted (1) between SP concentrations taken from the jugular and tail vein (at 08:00 a.m., TV1), (2) between the blood plasma of the jugular and tail vein (TV1) and the age, days in milk, lactation, and heart rate of animals, and (3) between the blood plasma of the jugular and tail vein (TV1) and the corresponding THI. For the determination of differences between SP concentrations in blood taken from the jugular vein and the tail vein a Mann–Whitney U Test (= Wilcoxon Rank Sum Test) was conducted. Friedman test was used for comparing the SP concentrations in the tail vein between five timepoints. The significant Friedman test was followed by the Durbin-Conover post-hoc analysis, in order to access the pairwise differences between particular timepoints, with Holm-Correction of *p*-values for multiple comparisons.

### Supplementary Information

**Additional file 1: Appendix 1. **Parameters and clinical findings in 52 healthy adult cattle of the German Simmental breed which were sampled to assess substance P concentrations. Cattle had to be healthy and pregnant (days 60 to 250 of lactation). According to their milk yield and experimental feeding, animals were either grouped in feeding group 1 or 2.

**Additional file 2: Appendix 2. **Laboratory findings and results of fecal examination in 52 healthy adult cattle of the German Simmental breed which were sampled to assess substance P concentrations, with reference ranges. Laboratory findings were within the reference range determined by the Clinic for Ruminants with Ambulatory and Herd Health Services in 21 animals (PHYS) and slightly below or above the references ranges in 31 animals (MDEV). Glutaraldehyde test is missing in one animal (28).

**Additional file 3: Appendix 3. **Substance P concentrations in 52 healthy adult cattle of the German Simmental Breed. Blood samples were taken at 07:45 a.m. from the Vena jugularis (BJV), at 08:00 a.m. from the Vena caudalis mediana (TV1), and repeatedly at 02:00 p.m. (TV2), 08:00 p.m. (TV3), 02:00 a.m. (TV4), and 08:00 a.m. (TV5). Substance P concentrations are given in pg/ml. Missing values are presented as nA (not applicable) – values are missing because blood sampling at the Vena caudalis mediana was not possible (*n* = 15) and because one sample was mislabeled and therefore excluded from the statistical analysis (*n* = 1). Number of animal is presented as Nr. A sensitivity of 167.78 pg/ml was defined in our laboratory.

## Data Availability

All data generated or analysed during this study are included in this published article and its supplementary information files.

## Abbreviations

BHBA: Beta Hydroxybutyrate
BJV: Blood Sample taken from the Jugular Vein at 07:45 a.m.
D: Day
Dl: Deciliter
EDTA: Ethylenediamine tetraacetic acid
ELISA: Enzyme-Linked Immunosorbent Assay
G: Gauge
G: Gramm
gHb: Gramm Hemoglobin
l: Liter
NEFAs: Non-esterified fatty acids
MDEV: Mild Deviations from Reference Ranges in Laboratory Findings
PHYS: Physiologic Laboratory Findings
PCV: Packed Cell Volume
PSPC: Plasma Substance P Concentration
Rho: Spearman Correlation Coefficient
SD: Standard Deviation
SP: Substance P
THI: Temperature Humidity Index
TV1: Blood Sample taken from the Tail Vein at 08:00 a.m. on Day 1
TV2: Blood Sample taken from the Tail Vein at 02:00 p.m. on Day 1
TV3: Blood Sample taken from the Tail Vein at 08:00 p.m. on Day 1
TV4: Blood Sample taken from the Tail Vein at 02:00 a.m. on Day 2
TV5: Blood Sample taken from the Tail Vein at 08:00 a.m. on Day 2
U: Unit
µl: Microliter
°C: Degree Celcius
