# Supplementary material for: Evaluation of Substance P concentrations in the blood plasma of jugular and tail vein of healthy German Simmental cows
Source: BMC Vet Res. 2023 Oct 20;19:218. doi: 10.1186/s12917-023-03768-0 (PMC10588027; doi:10.1186/s12917-023-03768-0)
Supplement: Supplementary file 1 — Additional file 1: Appendix 1. Parameters and clinical findings in 52 healthy adult cattle of the German Simmental breed which were sampled to assess substance P concentrations. Cattle had to be healthy and pregnant (days 60 to 250 of lactation). According to their milk yield and experimental feeding, animals were either grouped in feeding group 1 or 2. [file 12917_2023_3768_MOESM1_ESM.docx]

**Appendix 1:** Parameters and clinical findings in 52 healthy adult cattle of the German Simmental breed which were sampled to assess substance P concentrations. Cattle had to be healthy and pregnant (days 60 to 250 of lactation). According to their milk yield and experimental feeding, animals were either grouped in feeding group 1 or 2.

| **Nr^1^** | **Age^2^** | **Lactation** | **Days PP^3^** | **Days pregnant** | **Milk (kg)^4^** | **T^5^** | **HR^6^** | **RR^7^** | **RP^8^** | **Faeces^9^** | **Group** |
| --- | --- | --- | --- | --- | --- | --- | --- | --- | --- | --- | --- |
| **1** | 6.4 | 4 | 186 | 84 | 34.1 | 38.2 | 84 | 36 | 2 | normal | 2 |
| **2** | 4.5 | 3 | 141 | 53 | 37.3 | 38.2 | 84 | 24 | 3 | normal | 2 |
| **3** | 3.7 | 3 | 178 | 54 | 34.4 | 38.3 | 84 | 28 | 2 | normal | 2 |
| **4** | 6.1 | 4 | 192 | 89 | 31.4 | 38.5 | 84 | 36 | 3 | normal | 1 |
| **5** | 5.5 | 4 | 132 | 45 | 45.6 | 38 | 80 | 28 | 3 | normal | 2 |
| **6** | 3.8 | 2 | 153 | 43 | 39.1 | 38.1 | 76 | 24 | 2 | normal | 2 |
| **8** | 4 | 2 | 239 | 166 | 35.1 | 38.1 | 84 | 32 | 3 | normal | 1 |
| **9** | 4.9 | 3 | 191 | 119 | 31.8 | 38.3 | 80 | 32 | 2 | slightly loose | 1 |
| **10** | 3.7 | 2 | 157 | 43 | 36.8 | 38.6 | 80 | 32 | 3 | normal | 1 |
| **11** | 6.1 | 4 | 238 | 166 | 31.9 | 38 | 76 | 36 | 2 | normal | 1 |
| **14** | 6.9 | 5 | 188 | 74 | 41.2 | 38.1 | 72 | 36 | 3 | slightly loose | 2 |
| **15** | 4.9 | 3 | 205 | 102 | 42.4 | 38 | 84 | 32 | 3 | slightly loose | 2 |
| **16** | 4.9 | 3 | 200 | 76 | 41 | 38.2 | 80 | 32 | 2 | slightly loose | 2 |
| **17** | 4.8 | 3 | 194 | 50 | 48.5 | 38 | 80 | 28 | 3 | normal | 2 |
| **18** | 3.9 | 2 | 186 | 43 | 40 | 38 | 76 | 28 | 3 | normal | 2 |
| **19** | 4.1 | 2 | 189 | 105 | 36.6 | 38.3 | 84 | 24 | 2 | normal | 2 |
| **20** | 5.5 | 4 | 129 | 51 | 42.9 | 38.2 | 80 | 20 | 3 | normal | 2 |
| **21** | 5.5 | 4 | 184 | 60 | 45.4 | 38.1 | 84 | 32 | 2 | normal | 2 |
| **24** | 6.2 | 4 | 210 | 75 | 31.9 | 38 | 80 | 40 | 3 | normal | 1 |
| **25** | 7.8 | 6 | 159 | 47 | 39.4 | 38.1 | 88 | 44 | 2 | normal | 2 |
| **28** | 5.3 | 3 | 180 | 40 | 45.6 | 38.2 | 84 | 40 | 3 | normal | 2 |
| **29** | 4.9 | 3 | 137 | 63 | 51.6 | 38.3 | 72 | 44 | 2 | normal | 1 |
| **30** | 3.5 | 2 | 141 | 46 | 37.5 | 38.9 | 88 | 44 | 3 | normal | 2 |
| **32** | 7.6 | 6 | 126 | 48 | 36.5 | 39 | 80 | 40 | 3 | normal | 1 |
| **34** | 5 | 3 | 120 | 39 | 38.4 | 38.9 | 84 | 48 | 2 | slightly loose | 1 |
| **35** | 4.9 | 3 | 216 | 74 | 38 | 39.1 | 80 | 40 | 3 | normal | 2 |
| **36** | 4.5 | 2 | 233 | 40 | 40.5 | 39 | 88 | 48 | 3 | normal | 1 |

Continuing Appendix 1:

| **Nr^1^** | **Age^2^** | **Lactation** | **Days PP^3^** | **Days pregnant** | **Milk (kg)^4^** | **T^5^** | **HR^6^** | **RR^7^** | **RP^8^** | **Faeces** | **Group** |
| --- | --- | --- | --- | --- | --- | --- | --- | --- | --- | --- | --- |
| **38** | 3.6 | 2 | 177 | 48 | 33.3 | 38.2 | 84 | 48 | 3 | normal | 2 |
| **41** | 3.9 | 2 | 202 | 126 | 35.1 | 38.3 | 80 | 40 | 3 | normal | 2 |
| **42** | 4.7 | 3 | 168 | 52 | 39.3 | 38.5 | 68 | 40 | 3 | normal | 2 |
| **43** | 5.8 | 4 | 142 | 68 | 41.1 | 38 | 88 | 44 | 3 | normal | 2 |
| **44** | 3.6 | 2 | 177 | 91 | 34 | 38.7 | 80 | 32 | 2 | normal | 2 |
| **45** | 9.1 | 7 | 233 | 68 | 37.5 | 38 | 92 | 28 | 2 | normal | 2 |
| **46** | 3.4 | 2 | 117 | 46 | 40.8 | 38.7 | 88 | 32 | 2 | normal | 1 |
| **47** | 5.6 | 4 | 164 | 47 | 32.7 | 38.6 | 80 | 24 | 2 | normal | 1 |
| **48** | 5.3 | 3 | 149 | 41 | 38.6 | 38.5 | 80 | 36 | 2 | normal | 1 |
| **49** | 3.6 | 2 | 133 | 60 | 37.5 | 38.2 | 80 | 32 | 2 | normal | 1 |
| **51** | 3.9 | 2 | 128 | 58 | 33.2 | 38.6 | 80 | 32 | 3 | normal | 2 |
| **52** | 4.8 | 3 | 173 | 50 | 35.1 | 38.3 | 76 | 32 | 2 | normal | 2 |
| **54** | 5.9 | 4 | 197 | 52 | 36.1 | 38.8 | 96 | 24 | 2 | normal | 2 |
| **55** | 5.2 | 3 | 205 | 41 | 29.5 | 38.4 | 80 | 28 | 1 | normal | 2 |
| **56** | 4.8 | 3 | 119 | 43 | 34.4 | 38.8 | 76 | 28 | 2 | normal | 2 |
| **57** | 5.7 | 4 | 170 | 46 | 31.7 | 38.5 | 80 | 32 | 1 | normal | 1 |
| **59** | 5 | 3 | 228 | 54 | 31.7 | 38.3 | 80 | 32 | 3 | normal | 2 |
| **60** | 3.8 | 2 | 186 | 47 | 35.2 | 38.1 | 100 | 24 | 1 | normal | 2 |
| **62** | 4.9 | 3 | 216 | 99 | 32.5 | 38.2 | 84 | 40 | 1 | normal | 2 |
| **63** | 5.5 | 4 | 156 | 65 | 38 | 38.5 | 76 | 40 | 2 | normal | 2 |
| **64** | 4 | 2 | 230 | 47 | 29.6 | 38.1 | 80 | 36 | 1 | normal | 2 |
| **66** | 9.1 | 7 | 143 | 62 | 32.6 | 38 | 76 | 36 | 2 | normal | 1 |
| **72** | 3.7 | 2 | 163 | 73 | 45.7 | 38.5 | 88 | 36 | 2 | normal | 1 |
| **73** | 3.6 | 2 | 150 | 42 | 39 | 38.5 | 96 | 44 | 1 | normal | 2 |
| **76** | 3.8 | 2 | 169 | 97 | 38.3 | 38.6 | 86 | 24 | 1 | normal | 1 |

^1^ Number of animal. ^2^ Age in years. ^3^ Days post-partum. ^4^ Milk yield per day in kilogram. ^5^ Rectal Temperature in °C. ^6^ Heart Rate. ^7^ Respiratory Rate. ^8^ Rumen Peristaltic in 2 minutes. ^9^ Consistency of Faeces.
