# Supplementary material for: Evaluation of Substance P concentrations in the blood plasma of jugular and tail vein of healthy German Simmental cows
Source: BMC Vet Res. 2023 Oct 20;19:218. doi: 10.1186/s12917-023-03768-0 (PMC10588027; doi:10.1186/s12917-023-03768-0)
Supplement: Supplementary file 2 — Additional file 2: Appendix 2. Laboratory findings and results of fecal examination in 52 healthy adult cattle of the German Simmental breed which were sampled to assess substance P concentrations, with reference ranges. Laboratory findings were within the reference range determined by the Clinic for Ruminants with Ambulatory and Herd Health Services in 21 animals (PHYS) and slightly below or above the references ranges in 31 animals (MDEV). Glutaraldehyde test is missing in one animal (28). [file 12917_2023_3768_MOESM2_ESM.docx]

**Appendix 2:** Laboratory findings and results of fecal examination in 52 healthy adult cattle of the German Simmental breed which were sampled to assess substance P concentrations, with reference ranges. Laboratory findings were within the reference range determined by the Clinic for Ruminants with Ambulatory and Herd Health Services in 21 animals (PHYS) and slightly below or above the references ranges in 31 animals (MDEV). Glutaraldehyde test is missing in one animal (28).

| **Nr^1^** | **Laboratory Findings** | | | | | | | | **Faecal Findings** | | | **Group** |
| --- | --- | --- | --- | --- | --- | --- | --- | --- | --- | --- | --- | --- |
|  | **Leuc^2^**  **4 – 10 x10³/µl** | **HB^3^**  **10 – 13 g/dl** | **PCV^4^**  **30 – 36%** | **GSPHX^5^**  **>250 g/Hb** | **TP^6^**  **40 – 80 g/l** | **NEFA^7^**  **< 0.39 mmol/l** | **BHB^8^**  **<1.2 mmol/l** | **Glutar^9^**  **>15 min** | **LW^10^** | **LF/RF^11^** | **GIW^12^** |  |
| **1** | 6.82 | 11.40 | 35.32 | 600 | 77.10 | 0.15 | 0.56 | 14 | 0 | 0 | 0 | MDEV |
| **2** | 6.23 | 8.60 | 28.94 | 611 | 75.20 | 0.10 | 0.55 | >16 | 0 | 0 | 0 | MDEV |
| **3** | 10.29 | 10.00 | 31.42 | 443 | 76.20 | 0.12 | 0.62 | >16 | 0 | 0 | 0 | MDEV |
| **4** | 5.21 | 10.00 | 32.96 | 482 | 75.30 | 0.10 | 0.59 | >16 | 0 | 0 | 0 | PHYS |
| **5** | 5.81 | 10.30 | 32.86 | 559 | 83.60 | 0.11 | 0.57 | 14 | 0 | 0 | 0 | MDEV |
| **6** | 8.99 | 10.90 | 34.95 | 622 | 71.90 | 0.10 | 0.66 | >16 | 0 | 0 | 0 | PHYS |
| **8** | 7.08 | 10.10 | 32.32 | 696 | 74.50 | 0.09 | 0.51 | >16 | 0 | 0 | 0 | PHYS |
| **9** | 6.52 | 12.20 | 36.77 | 557 | 70.00 | 0.10 | 0.84 | >16 | 0 | 0 | 0 | MDEV |
| **10** | 5.68 | 9.00 | 27.93 | 598 | 72.30 | 0.10 | 0.83 | >16 | 0 | 0 | 0 | MDEV |
| **11** | 6.75 | 10.00 | 30.54 | 647 | 71.80 | 0.12 | 0.54 | >16 | 0 | 0 | 0 | PHYS |
| **14** | 5.92 | 10.90 | 35.61 | 423 | 81.00 | 0.09 | 0.69 | >16 | 0 | 0 | 0 | MDEV |
| **15** | 6.03 | 10.60 | 33.36 | 547 | 74.90 | 0.10 | 0.42 | >16 | 0 | 0 | 0 | PHYS |
| **16** | 6.51 | 9.70 | 30.24 | 510 | 68.00 | 0.10 | 0.42 | >16 | 0 | 0 | 0 | MDEV |
| **17** | 6.21 | 10.00 | 33.53 | 664 | 75.40 | 0.16 | 0.64 | >16 | 0 | 0 | 0 | PHYS |
| **18** | 5.70 | 9.60 | 31.25 | 821 | 71.30 | 0.30 | 0.46 | >16 | 0 | 0 | 0 | MDEV |
| **19** | 7.51 | 10.60 | 33.11 | 696 | 73.50 | 0.15 | 0.47 | >16 | 0 | 0 | 0 | PHYS |
| **20** | 6.71 | 10.00 | 33.59 | 367 | 78.30 | 0.11 | 0.36 | >16 | 0 | 0 | 0 | PHYS |
| **21** | 5.78 | 11.40 | 35.53 | 477 | 72.50 | 0.15 | 0.61 | >16 | 0 | 0 | 0 | PHYS |
| **24** | 7.82 | 11.30 | 37.16 | 530 | 67.30 | 0.10 | 0.87 | >16 | 0 | 0 | 0 | MDEV |
| **25** | 4.00 | 10.50 | 36.02 | 496 | 75.80 | 0.15 | 0.83 | >16 | 0 | 0 | 0 | MDEV |
| **28** | 6.7 | 8.4 | 27.85 | 587 | 72.80 | 0.08 | 0.78 | - | 0 | 0 | 0 | MDEV |

**Continuing Appendix 2:**

| **Nr^1^** | **Laboratory Findings** | | | | | | | | **Faecal Findings** | | | **Group** |
| --- | --- | --- | --- | --- | --- | --- | --- | --- | --- | --- | --- | --- |
|  | **Leuc^2^**  **4 – 10 x10³/µl** | **HB^3^**  **10 – 13 g/dl** | **PCV^4^**  **30 – 36%** | **GSPHX^5^**  **>250** | **TP^6^**  **40 – 80 g/l** | **NEFA^7^**  **< 0.39 mmol/l** | **BHB^8^**  **<1.2 mmol/l** | **Glutar^9^**  **>15 min** | **LW^10^** | **LF/RF^11^** | **GIW^12^** |  |
| **29** | 4.49 | 8.30 | 27.52 | 719 | 73.10 | 0.06 | 0.98 | >16 | 0 | 0 | 0 | MDEV |
| **30** | 7.95 | 9.90 | 30.19 | 623 | 71.10 | 0.14 | 0.70 | >16 | 0 | 0 | 0 | MDEV |
| **32** | 6.13 | 9.60 | 28.96 | 478 | 78.80 | 0.11 | 0.77 | >16 | 0 | 0 | 0 | MDEV |
| **34** | 6.90 | 9.50 | 29.57 | 514 | 78.80 | 0.12 | 0.78 | >16 | 0 | 0 | 0 | MDEV |
| **35** | 8.65 | 10.20 | 30.71 | 555 | 71.40 | 0.13 | 0.75 | >16 | 0 | 0 | 0 | PHYS |
| **36** | 4.84 | 9.90 | 30.33 | 667 | 76.40 | 0.15 | 0.73 | >16 | 0 | 0 | 0 | MDEV |
| **38** | 8.19 | 12.50 | 36.16 | 684 | 80.80 | 0.15 | 0.35 | >16 | 0 | 0 | 0 | MDEV |
| **41** | 5.52 | 12.00 | 35.76 | 598 | 67.50 | 0.11 | 0.34 | >16 | 0 | 0 | 0 | PHYS |
| **42** | 10.25 | 11.50 | 35.45 | 651 | 73.50 | 0.14 | 0.40 | >16 | 0 | 0 | 0 | MDEV |
| **43** | 7.31 | 10.80 | 33.61 | 512 | 77.70 | 0.10 | 0.52 | >16 | 0 | 0 | 0 | PHYS |
| **44** | 5.39 | 10.10 | 30.12 | 573 | 77.60 | 0.07 | 0.67 | >16 | 0 | 0 | 0 | PHYS |
| **45** | 8.36 | 11.20 | 34.93 | 661 | 74.30 | 0.10 | 0.78 | >16 | 0 | 0 | 0 | PHYS |
| **46** | 7.52 | 8.70 | 26.53 | 559 | 67.00 | 0.07 | 0.62 | >16 | 0 | 0 | 0 | MDEV |
| **47** | 3.96 | 9.40 | 29.10 | 521 | 67.10 | 0.03 | 0.84 | >16 | 0 | 0 | 0 | MDEV |
| **48** | 7.90 | 9.40 | 29.72 | 526 | 73.30 | 0.09 | 0.57 | >16 | 0 | 0 | 0 | MDEV |
| **49** | 8.75 | 10.00 | 30.15 | 766 | 78.90 | 0.07 | 0.46 | >16 | 0 | 0 | 0 | PHYS |
| **51** | 6.71 | 10.80 | 33.53 | 559 | 74.80 | 0.06 | 0.82 | >16 | 0 | 0 | 0 | PHYS |
| **52** | 9.87 | 10.10 | 31.75 | 602 | 77.20 | 0.07 | 0.53 | >16 | 0 | 0 | 0 | PHYS |
| **54** | 12.71 | 12.00 | 36.78 | 643 | 79.80 | 0.10 | 0.56 | >16 | 0 | 0 | 0 | MDEV |
| **55** | 7.56 | 10.80 | 34.20 | 766 | 76.30 | 0.14 | 0.60 | >16 | 0 | 0 | 0 | PHYS |
| **56** | 4.50 | 10.60 | 34.40 | 747 | 75.50 | 0.10 | 0.55 | >16 | 0 | 0 | 0 | PHYS |
| **57** | 9.96 | 12.00 | 37.02 | 703 | 82.30 | 0.11 | 0.43 | >16 | 0 | 0 | 0 | MDEV |
| **59** | 7.60 | 13.60 | 41.61 | 571 | 75.50 | 0.10 | 0.82 | >16 | 0 | 0 | 0 | MDEV |
| **60** | 7.50 | 9.50 | 29.65 | 614 | 79.60 | 0.13 | 0.79 | >16 | 0 | 0 | 0 | MDEV |
| **62** | 6.26 | 10.60 | 33.91 | 828 | 72.30 | 0.17 | 0.47 | >16 | 0 | 0 | 0 | PHYS |
| **63** | 5.38 | 10.60 | 32.06 | 802 | 73.90 | 0.15 | 0.85 | >16 | 0 | 0 | 0 | PHYS |
| **64** | 8.46 | 8.90 | 27.78 | 813 | 76.40 | 0.11 | 0.60 | >16 | 0 | 0 | 0 | MDEV |

**Continuing Appendix 2:**

| **Nr^1^** | **Laboratory Findings** | | | | | | | | **Faecal Findings** | | | **Group** |
| --- | --- | --- | --- | --- | --- | --- | --- | --- | --- | --- | --- | --- |
|  | **Leuc^2^**  **4 – 10 x10³/µl** | **HB^3^**  **10 – 13 g/dl** | **PCV^4^**  **30 – 36%** | **GSPHX^5^**  **>250** | **TP^6^**  **40 – 80 g/l** | **NEFA^7^**  **< 0.39 mmol/l** | **BHB^8^**  **<1.2 mmol/l** | **Glutar^9^**  **>15 min** | **LW^10^** | **LF/RF^11^** | **GIW^12^** |  |
| **66** | 6.67 | 10.80 | 31.94 | 501 | 81.00 | 0.10 | 0.51 | >16 | 0 | 0 | 0 | MDEV |
| **72** | 7.38 | 9.5 | 32.58 | 641 | 73.8 | 0.12 | 0.41 | >16 | 0 | 0 | 0 | MDEV |
| **73** | 6.97 | 9.3 | 29.96 | 799 | 75 | 0.1 | 0.59 | >16 | 0 | 0 | 0 | MDEV |
| **76** | 6.35 | 9.2 | 29.81 | 590 | 68.5 | 0.07 | 0.45 | >16 | 0 | 0 | 0 | MDEV |

^1^ Number of animal. ^2^ Leucocyte count. ^3^ Haemoglobin. ^4^ Packed Cell Volume. ^5^ Glutathione Peroxidase. ^6^ Total Protein. ^7^ Non-esterified Fatty Acids. ^8^ Beta hydroxybutyrate. ^9^ Glutaraldehyde test. ^10^ Lung worms. ^11^ Liver Fluke/Rumen Fluke. ^12^ Gastrointestinal Worms
