# Supplementary material for: Evaluation of Substance P concentrations in the blood plasma of jugular and tail vein of healthy German Simmental cows
Source: BMC Vet Res. 2023 Oct 20;19:218. doi: 10.1186/s12917-023-03768-0 (PMC10588027; doi:10.1186/s12917-023-03768-0)
Supplement: Supplementary file 3 — Additional file 3: Appendix 3. Substance P concentrations in 52 healthy adult cattle of the German Simmental Breed. Blood samples were taken at 07:45 a.m. from the Vena jugularis (BJV), at 08:00 a.m. from the Vena caudalis mediana (TV1), and repeatedly at 02:00 p.m. (TV2), 08:00 p.m. (TV3), 02:00 a.m. (TV4), and 08:00 a.m. (TV5). Substance P concentrations are given in pg/ml. Missing values are presented as nA (not applicable) – values are missing because blood sampling at the Vena caudalis mediana was not possible (n = 15) and because one sample was mislabeled and therefore excluded from the statistical analysis (n = 1). Number of animal is presented as Nr. A sensitivity of 167.78 pg/ml was defined in our laboratory. [file 12917_2023_3768_MOESM3_ESM.docx]

**Appendix 3:** Substance P concentrations in 52 healthy adult cattle of the German Simmental Breed. Blood samples were taken at 07:45 a.m. from the Vena jugularis (BJV), at 08:00 a.m. from the Vena caudalis mediana (TV1), and repeatedly at 02:00 p.m. (TV2), 08:00 p.m. (TV3), 02:00 a.m. (TV4), and 08:00 a.m. (TV5). Substance P concentrations are given in pg/ml. Missing values are presented as nA (not applicable) – values are missing because blood sampling at the Vena caudalis mediana was not possible (n = 15) and because one sample was mislabeled and therefore excluded from the statistical analysis (n = 1). Number of animal is presented as **Nr**. A sensitivity of 167.78 pg/ml was defined in our laboratory.

| **Nr** | **BJV** | **TV1** | **TV2** | **TV3** | **TV4** | **TV5** |
| --- | --- | --- | --- | --- | --- | --- |
| **1** | 1,313.718 | 986.821 | 1,250.189 | 167.78 | 1,406.736 | 1,239.073 |
| **2** | 762.157 | 753.871 | 612.241 | 810.982 | 529.244 | 694.006 |
| **3** | 1,285.616 | 850.275 | 815.547 | 928.118 | 851.276 | 841.659 |
| **4** | 1,000.833 | 791.7 | 777.886 | 910.706 | 921.768 | nA |
| **5** | 807.391 | 686.294 | 775.811 | nA | nA | nA |
| **6** | 822.532 | 712.212 | 636.492 | 167.78 | nA | 762.353 |
| **8** | 863.759 | 830.89 | 799.034 | 1,117.297 | 774.924 | 826.697 |
| **9** | 810.202 | 835.11 | 853.955 | 594.835 | nA | 961.563 |
| **10** | 886.639 | 1,150.535 | 1,013.85 | 1,057.937 | 1,001.666 | 883.198 |
| **11** | 653.789 | 636.171 | 591.078 | 999.195 | 1,135.861 | 457.729 |
| **14** | 1,770.433 | 1,798.15 | 1,540.956 | 1,543.519 | 1,411.483 | 2,244.764 |
| **15** | 618.173 | 635.419 | 713.66 | 728.967 | 748.086 | 465.258 |
| **16** | 1,883.115 | 2,009.852 | 2,511.615 | 2,292.837 | 2,973.533 | 3,359.525 |
| **17** | 958.23 | 985.075 | 1,049.532 | nA | 1,440.291 | 1,280.48 |
| **18** | 866.275 | 753.9 | 945.196 | 1,122.959 | 1,314.433 | 1,219.405 |
| **19** | 1,476.572 | 1,390.16 | nA | nA | 1,694.94 | 1,497.313 |
| **20** | 782.898 | 655.344 | 738.424 | 1,314.433 | 1,472.474 | 1,200.998 |
| **21** | 822.237 | 779.601 | 953.857 | 945.196 | 1,162.475 | 1125.717 |
| **24** | 842.024 | 792.905 | 789.55 | 877.799 | 1,238.225 | 860.593 |
| **25** | 2,337.251 | 1,083.71 | 1,295.841 | 1227.09 | 1,364.55 | nA |
| **28** | 2,201.708 | 2,531.002 | 2,197.662 | 2,277.713 | 2,378.626 | 2,094.688 |
| **29** | 960.871 | 995.17 | 1,037.327 | 1452.43 | 1,175.031 | nA |
| **30** | 865.466 | 779.591 | 713.937 | 900.11 | 1,019.921 | 983.859 |
| **32** | 1,005.721 | 725.888 | 660.377 | 867.259 | 842.838 | 849.295 |
| **34** | 1,001.048 | 856.069 | 947.771 | 559.097 | 940.311 | 962.636 |
| **35** | 699.873 | 515.752 | 579.92 | 780.673 | 1,074.474 | 811.509 |
| **36** | 757.217 | 736.499 | 803.816 | 692.851 | 808.477 | 928.775 |
| **38** | 1,060.564 | 952.839 | 1,252.19 | 1,305.871 | 1,301.569 | 1,334.023 |

**Continuing Appendix 3:**

| **Nr** | **BJV** | **TV1** | **TV2** | **TV3** | **TV4** | **TV5** |
| --- | --- | --- | --- | --- | --- | --- |
| **41** | 2,092.049 | 765.729 | 969.834 | 769.371 | 1,653.725 | 1,248.104 |
| **42** | 1,072.582 | 940.07 | 756.705 | 980.333 | 1,052.393 | 1,148.554 |
| **43** | 855.574 | 805.732 | 762.106 | 960.128 | 937.474 | 1,672.67 |
| **44** | 2,018.644 | 1,560.134 | 1,429.133 | 1,297.046 | 839.362 | 1,812.525 |
| **45** | 1,079.641 | 797.109 | 819.387 | 793.58 | 860.275 | 843.875 |
| **46** | 1,180.331 | 978.671 | 1,151.689 | 1,354.438 | 1,174.756 | 1,352.452 |
| **47** | 697.485 | 714.186 | 660.431 | 852.984 | 1,023.207 | 659.281 |
| **48** | 1,303.342 | 1,174.201 | 1,243.832 | 1,430.551 | 1,345.203 | 1,485.807 |
| **49** | 1,215.622 | 1,157.677 | 1,085.182 | 1,242.641 | 1,407.372 | 1,185.938 |
| **51** | 973.765 | 409.187 | 890.295 | 564.14 | 793.163 | 948.157 |
| **52** | 1,166.904 | 1,417.346 | 1,180.294 | 1,204.742 | 1,274.242 | 1,230.585 |
| **54** | 1,191.53 | 849.198 | 1,390.567 | 1,400.421 | nA | 952.431 |
| **55** | 999.949 | 764.342 | nA | 1,111.781 | 1,023.808 | 866.586 |
| **56** | 738.725 | 303.492 | 2,195.972 | 419.043 | 414.848 | 648.444 |
| **57** | 1,075.103 | 1,039.203 | 888.738 | 1,274.242 | 980.452 | 858.386 |
| **59** | 958.894 | 192.335 | 621.237 | 720.043 | 480.615 | 538.844 |
| **60** | 1,271.439 | 1,085.765 | 1,026.869 | 1,713.356 | 1,986.096 | 1,384.599 |
| **62** | 1,986.096 | 1,384.599 | 600.524 | 582.186 | 879.741 | 500.313 |
| **63** | 508.187 | 610.2 | 600.02 | 483.971 | 973.454 | 566.369 |
| **64** | 1,051.222 | 890.244 | 921.453 | 991.939 | 1,448.74 | 771.488 |
| **66** | 727.183 | 833.242 | 686.817 | nA | 1,080.515 | 935.612 |
| **72** | 501.948 | 723.956 | 496.872 | 743.307 | 811.353 | 760.423 |
| **73** | 993.801 | 594.939 | 791.206 | 969.396 | 830.533 | 937.355 |
| **76** | 761.463 | 650.97 | 739.933 | 570.764 | nA | nA |
